# Genome-Wide Analysis of Glycerol-3-Phosphate Acyltransferase (GPAT) Family in *Perilla frutescens* and Functional Characterization of *PfGPAT9* Crucial for Biosynthesis of Storage Oils Rich in High-Value Lipids

**DOI:** 10.3390/ijms242015106

**Published:** 2023-10-12

**Authors:** Yali Zhou, Xusheng Huang, Ting Hu, Shuwei Chen, Yao Wang, Xianfei Shi, Miao Yin, Runzhi Li, Jiping Wang, Xiaoyun Jia

**Affiliations:** 1 College of Agronomy/Institute of Molecular Agriculture & Bioenergy, Shanxi Agricultural University, Jinzhong 030801, China; zhouylnd@163.com (Y.Z.); wangyao04051015@163.com (Y.W.); jiaxiaoyun@sxau.edu.cn (X.J.); 2 College of Life Sciences, Shanxi Agricultural University, Jinzhong 030801, China

**Keywords:** Perilla (*Perilla frutescens*), glycerol-3-phosphate acyltransferase (GPAT), polyunsaturated fatty acid (PUFA), triacylglycerol (TAG) biosynthesis

## Abstract

Glycerol-3-phosphate acyltransferase (GPAT) catalyzes the first step in triacylglycerol (TAG) biosynthesis. However, GPAT members and their functions remain poorly understood in *Perilla frutescens*, a special edible-medicinal plant with its seed oil rich in polyunsaturated fatty acids (mostly α-linolenic acid, ALA). Here, 14 PfGPATs were identified from the *P. frutescens* genome and classified into three distinct groups according to their phylogenetic relationships. These 14 *PfGPAT* genes were distributed unevenly across 11 chromosomes. PfGPAT members within the same subfamily had highly conserved gene structures and four signature functional domains, despite considerable variations detected in these conserved motifs between groups. RNA-seq and RT-qPCR combined with dynamic analysis of oil and FA profiles during seed development indicated that PfGPAT9 may play a crucial role in the biosynthesis and accumulation of seed oil and PUFAs. Ex vivo enzymatic assay using the yeast expression system evidenced that PfGPAT9 had a strong GPAT enzyme activity crucial for TAG assembly and also a high substrate preference for oleic acid (OA, C18:1) and ALA (C18:3). Heterogeneous expression of *PfGPAT9* significantly increased total oil and UFA (mostly C18:1 and C18:3) levels in both the seeds and leaves of the transgenic tobacco plants. Moreover, these transgenic tobacco lines exhibited no significant negative effect on other agronomic traits, including plant growth and seed germination rate, as well as other morphological and developmental properties. Collectively, our findings provide important insights into understanding PfGPAT functions, demonstrating that PfGPAT9 is the desirable target in metabolic engineering for increasing storage oil enriched with valuable FA profiles in oilseed crops.

## 1. Introduction

Vegetable oils serve as essential components in the daily diet of humans, supplying approximately one-fourth of the dietary energy. Moreover, vegetable oils are valuable sources of carbon-neutral fuels and renewable raw materials for various industrial products, such as detergents, lubricants, and nylon [1,2,3,4]. The biosynthesis and accumulation of storage oils (mostly in the form of triacylglycerol (TAG)) primarily occurred in plant seeds, providing a carbon source and energy reservoir for seed germination and seedling growth. In addition, a few oil plants can enrich a substantial amount of storage oils in other organs, such as in the fruit pulps of *Trachycarpus fortune* and *Canarium album* [5] and in the underground tubers of *Cyperus esculentus* [6]. To meet a globally growing demand for vegetable oils, more efforts are needed to investigate the molecular mechanisms underlying FA and oil biosynthesis and accumulation in oleogenic plants, thus increasing oil yields and the production of valuable fatty acids (FAs).

TAG is comprised of three fatty acid moieties linked to the glycerol backbone through ester linkages, being present in nearly all plant tissues/organs [7,8,9] and playing diverse biological functions [10]. According to the presence and number of double bonds within FA chains incorporated into TAG, TAG-associated FAs in cells can be classified into saturated FAs (SFAs), monounsaturated FAs (MUFAs), and polyunsaturated FAs (PUFAs). The difference in the three FAs attached to glycerol determines the chemical properties and physiological functions of TAGs and the commercial applications of TAGs [11]. For example, TAGs rich in SFAs have superior oxidative stability but fewer health benefits. TAGs enriched with MUFAs (e.g., oleic acid, OA, C18:1) demonstrate high oxidation stability and are desirable oils for human health, the food industry, and biofuel production [12]. TAGs bearing PUFAs such as α-linolenic acid (ALA, C18:3), eicosapentaenoic acid (EPA, C20:5), and docosahexaenoic acid (DHA, C22:6) display high oxidation instability but more health benefits to humans in both reducing the risk of multiple diseases and promoting brain development [13,14]. In view of this, an emerging research hotspot is to develop engineering plants for the commercial production of high-value vegetable oils enriched with rationally designed SFA:MUFA:PUFA profiles. Such customized oils are desirable for distinct applications in foods, pharmaceuticals, or other industries [15,16,17].

Oil biosynthesis in plant tissues involves de novo FA synthesis in the plastid, TAG assembly in the endoplasmic reticulum (ER), and oil body formation, which is controlled by a complex network of genes and enzymes [18,19]. The TAG assembly in the Kennedy pathway from glycerol-3-phosphate (G3P) and acyl-CoAs involves a series of enzymatic steps catalyzed by a group of enzymes [1,20,21,22]. Firstly, the glycerol-3-phosphate acyltransferase (GPAT) transfers an FA from acyl-CoA to the hydroxyl group at the *sn*-1 position of G3P, resulting in the formation of lysophosphatidic acid (LPA). Then, the lysophosphatidic acid acyltransferase (LPAAT) esterifies a second FA to the hydroxyl group at the *sn*-2 position of LPA to generate phosphatidic acid (PA). Thirdly, the phosphatidic acid phosphatase (PAP) facilitates the hydrolysis of the sn-3 phosphate group to produce de novo diacylglycerol (DAG). In the final step of TAG biosynthesis, diacylglycerol acyltransferase (DGAT) or phospholipid:diacylglycerol acyltransferase (PDAT) catalyzes a third FA from Acyl-CoA or phosphatidylcholine (PC) to incorporate onto the *sn*-3 position of DAG to generate TAG [23]. TAG continuously accumulates in the ER and specifically binds to oleosin and caleosin, resulting in the formation of mature oil bodies that are stored in the ER or further released into the cytoplasm. These oil bodies provide essential nutrient and energy reserves for plant seed germination and early seedling growth [24,25,26].

As the first acyl-esterifying enzyme in the biosynthesis of glycerolipids, including storage TAGs, various polar membrane lipids, and numerous signaling molecules, GPAT (EC 2.3.1.15) was examined to be involved in different metabolic pathways and physiological functions associated with plant growth, development, and resistance to biotic and abiotic stress. Therefore, GPAT has been considered a potential genetic manipulation target for biotechnological applications. To date, GPAT genes have been identified in the model plant *Arabidopsis thaliana* [27,28,29] and a variety of other plant species, such as tomato (*Lycopersicum esculentum*) [30], spinach (*Spinacia oleracea*) [31], sunflower (*Helianthus annuus*) [32], rice (*Oryza sativa*) [33], and cotton (*Gossypium sp.*) [34]. According to their subcellular locations, GPATs can be classified into three different types: a soluble isoform located in the plastidial stroma using acyl-ACP as its natural acyl substrate, and two membrane-bound isoforms that exist in the ER and mitochondria with acyl-CoA and acyl-ACP as natural acyl donors, respectively [35].

Ten GPAT members have been identified in *Arabidopsis*, including ATS1 and AtGPAT1-9 [27,28,36,37]. Among them, ATS1 is located in the plastids, AtGPAT1/2/3 is localized in mitochondria, and AtGPAT4/5/6/7/8/9 is located in the ER [27,28,33]. ATS1 and AtGPAT9 were identified to possess *sn*-1 acyltransferase activity, while AtGPAT1-8 exhibited *sn*-2 acyltransferase activity, and AtGPAT4/6/8 acted as the bifunctional *sn*-2 acyltransferase/phosphatase [38]. Different enzymatic features and physiological functions have been found for these *Arabidopsis* GPAT members. For example, ATS1, using acyl-ACP as its substrate, is a crucial enzyme required for the biosynthesis of phosphatidylglycerols that make up plastidial membranes in the chloroplasts [36,39]. AtGPAT1, affecting *Arabidopsis* seed setting rate, displays *sn*-2 acyltransferase activity but no phosphatase activity, with dicarboxylic acyl-CoA as its substrate to generate LPA, likely involving the biosynthesis of extracellular lipids [29]. At the same time, no detectable acyltransferase activity is observed for AtGPAT2 and AtGPAT3 [29]. AtGPAT4 is potentially involved in the synthesis of extracellular cutin polyester in the stems and leaves of *Arabidopsis*, while AtGPAT5 is associated with the synthesis of extracellular suberin polyester in the root and seed coat [37,40]. Both AtGPAT6 and AtGPAT8 contribute to the cutin synthesis in the flower petals [41]. The *Atgpat6* T-DNA mutant and overexpression *Arabidopsis* lines showed a strong reduction and enhancement in cutin content on the petal surfaces, respectively. AtGPAT7 plays a role in the synthesis of cork tissue [27,29]. Moreover, AtGPAT1-3 exhibits distinct phylogenetic relationships compared to AtGPAT4-8 [38].

In particular, AtGPAT9, a homologous protein to microsomal GPAT responsible for storage oil production in mammalian cells, was recently characterized to play a crucial role in the biosynthesis of membrane lipids and storage oils, also facilitating lipid droplet formation during pollen grain maturation in *Arabidopsis* [42,43]. For its biochemical property, AtGPAT9 has *sn*-1 acyltransferase activity with a substrate preference toward acyl-CoAs, as revealed by an enzymatic assay of the microsomal fractions derived from yeast cells expressing *AtGPAT9* [43,44]. The knockdown of seed-specific *AtGPAT9* in *Arabidopsis* resulted in a decrease of 26% to 44% in total oil content in the seeds compared to the wild-type plant, suggesting that AtGPAT9 functions importantly in TAG synthesis in seeds [42]. Furthermore, loss-of-function mutant Atgpat9 exhibited a lethal embryo phenotype in *Arabidopsis*, indicating that AtGPAT9 plays a critical role in membrane lipid synthesis [42]. However, overexpression of AtGPAT9 significantly increased polar lipid and TAG levels in the leaves and greatly enhanced the number of lipid droplets in the pollen grains of Arabidopsis [42,43]. Similarly, overexpression of peanut *AhGPAT9* (*AtGPAT9* homolog) also significantly improved the seed oil content of *A. thaliana* [45]. Moreover, overexpression of *AtGPAT9*-type *GPAT* largely promoted TAG accumulation in red alga (*Cyanidioschyzon merolae*) [46]. The GPAT9 homolog from the oleaginous green microalga *Lobosphaera incisa* enhanced TAG levels by up to 50% when heterologously expressed in *Chlamydomonas reinhardtii* [47]. Heterologous expression of a yeast *GPAT* (*SCT1*) from *Arxula adeninivorans* led to a very high level of accumulation of oleates (>90%) in the oleaginous yeast *Yarrowia lipolytica* without growth panel, indicating the promising value of GPAT in modifying fatty acid profiles via biotechnology [48]. Collectively, these known reports demonstrate that GPAT9 can serve as a novel genetic resource for enhancing the yield of storage oil in seeds or vegetative tissues and improving fatty acid profiles and plant stress resistance [42] in addition to other GPATs. Therefore, identifying genes encoding GPAT involved in plant glycerolipid biosynthesis from a variety of plant species is crucial for elucidating the GPAT-mediated different metabolic pathways and physiological functions, providing new knowledge for the development of engineered plant oils containing desired nutritional or industrial properties.

Perilla (*Perilla frutescens* var. *frutescens*, 2n = 40), a recently identified allopolyploid annual herbaceous species belonging to the *Lamiaceae* family [49,50,51], is currently extensively cultivated in various East Asian countries, including China, Japan, and Korea [52]. As a traditional medicinal and edible plant, perilla is widely utilized in the pharmaceutical, healthcare, functional food development, and cosmetic industries [53,54]. Particularly, perilla is also an important oil crop with a high seed oil content of up to 60% (*w*/*w*), and the seed oil contains over 65% of ALA [55]. Perilla is recognized as one of the plants with the highest content of ALA [50]. Numerous studies have shown that ALA, an essential ω-3 FA for human health, has a unique effect on protecting the cardiovascular system and promoting human brain development [56]. Therefore, high-quality perilla seed oil can serve as a desirable ALA source for human nutrition [50]. In comparison to many studies focusing on the identification of perilla metabolites and their biological activities [57,58], limited work is currently being conducted to investigate the molecular mechanisms of FA (especially PUFA) and TAG biosynthesis in perilla seeds [50]. The recently published *P. frutescens* genome provides an important dataset source for the identification and functional analysis of crucial genes responsible for FA and TAG biosynthesis in perilla seed [51]. However, no GPAT was identified from perilla, despite a number of GPATs being reported in various plant species, such as *Arabidopsis* [27], cotton [34], and oil palm [59].

In this study, a genome-wide identification was performed for the GPAT family in perilla, followed by comprehensive analyses of gene structure, conserved motif, chromosome distribution, phylogeny, and expression patterns. Furthermore, the expression profiles of the *PfGPAT9* gene in various tissues and developing seeds were examined using real-time quantitative PCR (RT-qPCR). Importantly, functions of PfGPAT9 in oil biosynthesis and UFA accumulation were identified by ex vivo enzyme assays using a yeast (*Saccharomyces cerevisiae*)-based expression system and ectopic expression of PfGPAT9 in the common tobacco plant. The results reveal that PfGPAT9 prefers acyl-CoA (C18:1 > C18:3) as substrates and produces *sn*-1 LPA subsequently into TAG assembly, indicating PfGPAT9 as a valuable target used for the enhanced production of storage oils in transgenic oilseed crops. Furthermore, the present findings yield a scientific basis for further elucidating the functions of PfGPATs and the molecular mechanism of intracellular and extracellular lipid biosynthesis in plants.

## 2. Results

### 2.1. Twenty-Four GPAT Family Members Were Identified in the P. frutescens Genome

A total of 14 PfGPAT proteins (all GPAT protein IDs were listed in Table S1) encoded by 14 *PfGPAT* genes were identified in the *P. frutescens* genome (https://www.ncbi.nlm.nih.gov/genbank (accessed on 6 January 2023)) [51] via BLAST analysis. We used the online bioinformatics tool ProtParam to predict the basic physical and chemical properties of the PfGPAT proteins. The detailed information on the *PfGPAT* genes is presented in Table S2, including their respective groups, gene locus ID, chromosomal location, genomic position, CDS length, amino acid number, molecular weight (MW), theoretical isoelectric point (pI), GRAVY score, instability index, and aliphatic index of the proteins.

All the *PfGPAT* genes differed in terms of CDS length, which ranged from 1116 bp (*PfGPAT9*) to 1509 bp (*PfGPAT6-3* and *PfGPAT6-4*). The encoded protein length, MW, and pI were also diverse. The longest proteins identified were PfGPAT6-3 and PfGPAT6-4, both consisting of 502 amino acids, whereas PfGPAT9 was the shortest protein, comprising only 371 amino acids. The MWs of 14 PfGPAT members ranged from 42.89 kDa to 56.75 kDa. The pIs of PfATS1-1 and PfATS1-2 proteins were < 7, indicating that they were acidic proteins. However, the remaining 12 PfGPAT proteins exhibited a pI > 7, suggesting that these members are basic proteins. According to the predicted total average hydrophilicity, four PfGPAT members (PfATS1-1, PfATS1-2, PfGPAT9, and PfGPAT6-3) are hydrophilic proteins, and the other members are all hydrophobic proteins. Moreover, we used the online bioinformatics tools PSORT II (http://psort.hgc.jp/form2.html (accessed on 7 January 2023)) and TMHMM (http://www.cbs.dtu.dk/services/TMHMM-2.0/ (accessed on 7 January 2023)) to predict the subcellular localization and transmembrane domains of each PfGPAT protein. They were predicted to be localized in the ER, mt, and chloroplast (cp), respectively. PfGPAT9, PfGPAT4, and PfGPAT8 contain three transmembrane domains, while PfATS1-1 and PfATS1-2 have no transmembrane domains. All other PfGPAT members exhibit two transmembrane domains (Table S2). The 14 *PfGPAT* genes were detected to have an uneven distribution across 11 chromosomes, except for chromosomes 01, 03, 07, 08, 09, 10, 13, 16, and 19 (Figure S1 and Table S2). Two *PfGPAT* genes were identified on Chromosomes 04, 06, and 12, while only one *PfGPAT* gene was observed on each of the chromosomes 02, 05, 11, 14, 15, 17, 18, and 20.

### 2.2. Phylogenetic Analysis and Multiple Sequence Alignment of PfGPAT Proteins

**Figure 1:**
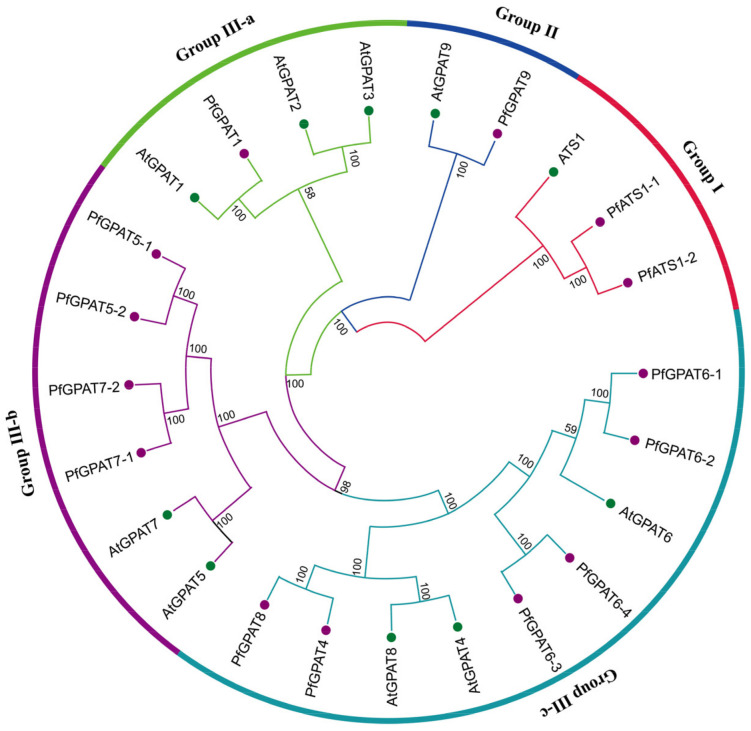
Phylogenetic relationships of GPAT proteins between *P. frutescens* and *Arabidopsis*. The colored regions represent different groups. The purple and green solid circles represent PfGPAT and AtGPAT proteins, respectively. The 14 PfGPAT proteins can be divided into three groups (I, II, and III), and group III can be further divided into three subgroups (III-a, III-b, and III-c).

**Figure 2:**
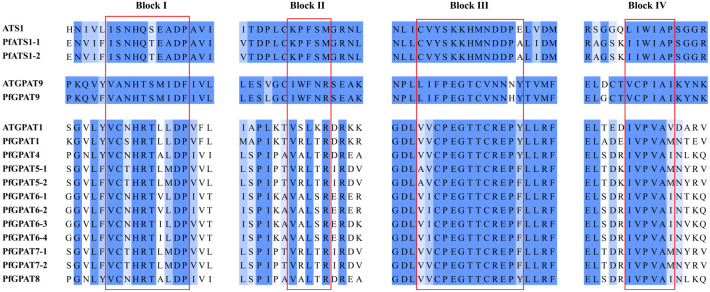
Alignment of perilla PfGPAT proteins and identification of the conserved amino acid motifs. The four conserved acyltransferase amino acid motifs (Blocks I–IV) are boxed.

In order to ascertain the evolutionary relationship of the 14 PfGPAT proteins and the ten *Arabidopsis* AtGPATs whose functions have been elucidated in detail, we constructed a phylogenetic tree (Figure 1) based on the multiple alignment of these GPAT protein sequences by using the MEGA 11.0.9 software with the neighbor-joining (N-J) method. Eventually, these 24 GPAT proteins were divided into three groups in the phylogenetic tree (Figure 2). Group I includes PfATS1-1/1-2 and AtATS1. Group II contains PfGPAT9 and AtGPAT9. Group III can be further classified into three subgroups. Subgroup III-a contains one PfGPAT (PfGPAT1) and three AtGPATs (AtGPAT1-3). Subgroup III-b consists of four PfGPATs (PfGPAT5-1, 5-2, 7-1, and 7-2) and two AtGPATs (AtGPAT5 and 7). Subgroup III-c includes six PfGPATs (PfGPAT4, 6-1, 6-2, 6-3, 6-4, and 8) and three AtGPATs (AtGPAT4, 6 and 8). Such phylogenetic analysis provides clues for the functional investigation of PfGPAT proteins in *P. frutescens*.

The alignment of GPAT proteins across evolutionarily diverse species has revealed the presence of four highly conserved amino acid motifs, which play a crucial role in both acyltransferase activity and G-3-P substrate binding [60]. A multiple sequence alignment of all the PfGPAT proteins was performed to identify the conserved domains within these proteins (Figure 2). The four highly conserved motifs were detected within each evolutionary clade (groups I, II, and III), respectively, with variations among the groups, indicating that PfGPAT proteins from different groups possess distinct evolutionary features.

### 2.3. Gene Structure and Conserved Motif Analysis Further Confirmed the Subgroup Classification

**Figure 3:**
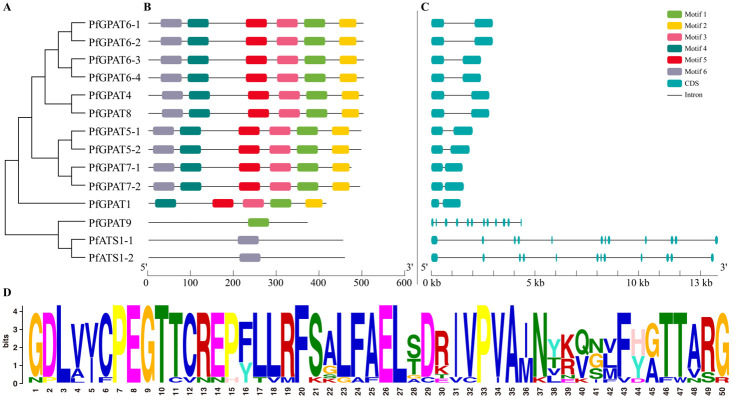
Analysis of the conserved motif and gene structure of the *P. frutescens* GPAT family. (**A**) The evolutionary tree of the *PfGPAT* gene family. (**B**) Motif analysis of the *PfGPAT* family. (**C**) The gene structure of *PfGPATs*. (**D**) GPAT conservative motif (Motif 1) of *P. frutescens*. The various color boxes represent different motifs or gene structures.

The MEME online tool was employed to identify the conserved motifs of *PfGPAT* gene family members, and a total of six conserved motifs were obtained. The conserved motifs and gene structure of the *PfGPAT* gene family were constructed based on the genome data of *P. frutescens* using TBtools 2.0 (Figure 3). As shown in the figure, the left side illustrates the phylogenetic relationships (Figure 3A), while the remaining sections represent the conserved motifs (Figure 3B) and gene structure (Figure 3C).

All six motifs examined (Motifs 1–6) are conserved among Group III *PfGPAT* members, except for Motif 6, which is absent in *PfGPAT1*. However, *PfGPAT9* (Group II) contains only Motif 1 (Figure 3D), while PfATS1-1 and PfATS1-2 (Group I) have only Motif 6. All the *PfGPAT*-encoding sequences were separated by introns. *PfATS1-1* and *PfATS1-2* from Group I and *PfGPAT9* from Group II contain 11 introns. Furthermore, a total of 11 *PfGPAT* genes in Group III only have one intron. In short, members from the same group exhibited similar conserved motifs and gene structures, further supporting the subgroup classification of PfGPATs based on the phylogenetic analysis.

### 2.4. Expression Pattern of PfGPAT9 Gene Is Consistent with Oil Accumulation during Developing Seeds of P. frutescens

**Figure 4:**
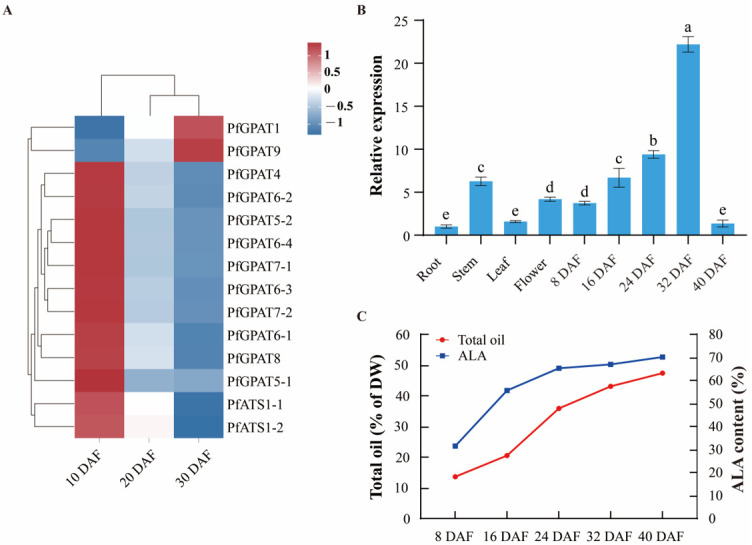
Analysis of *PfGPAT* gene expression patterns and the dynamic accumulation of total oil in seeds at different stages of the development of *P. frutescens*. (**A**) Heat map of transcriptome expression profiles of the 14 *PfGPAT* genes in different development stages of *P. frutescens* seeds. (**B**) The relative expression pattern of the *PfGPAT9* gene in various tissues and different developing seeds of *P. frutescens*. Values are presented as mean ± SD (*n* = 3). Different lowercase letters indicate significant differences at *p* < 0.05. (**C**) Total oil and ALA dynamic accumulation during seed development of *P. frutescens*. Total oils were extracted from samples of different developing stages of seeds. The total oil and ALA contents were determined by the methods described in the Section 4.

Our transcriptome data showed that the 14 *PfGPAT* genes were expressed in developing seeds, with *PfGPAT9* having a much higher level (Figure 4A). In order to further detect the expression pattern of the *PfGPAT9* gene, RT-qPCR was performed to examine its expression profile in various tissues and different developmental stages of *P. frutescens* seeds. The relative expression level of the *PfGPAT9* gene was determined by RT-qPCR using the *P. frutescens Actin* gene as the internal control. The *PfGPAT9* gene was expressed in root, stem, leaf, flower, and seed, but the expression levels in each tissue/organ were significantly different (Figure 4B). The expression of the *PfGPAT9* gene gradually increased from 8 DAF (days after flowering) to 24 DAF, followed by a rapid enhancement to the peak level from 24 DAF to 32 DAF (the expression level at 32 DAF exhibited a 4.93-fold increase compared to that at 8 DAF), and subsequently reduced sharply to a very low expression level at 40 DAF. The expression of the *PfGPAT9* gene exhibited lower levels in the root and leaf but much higher levels in the flower and developing seeds, suggesting that the *PfGPAT9* gene may play a significant role in the oil biosynthesis of *P. frutescens* seeds.

The present study showed that the total oil accumulation patterns in perilla (variety ‘Jinzisu 1’) seeds could be briefly divided into three key stages (Figure 4C). Total oil started to accumulate at a very small level of 13.6% (of seed dry weight, DW) in perilla seed at 8 DAF. It increased rapidly to 35.9% from 16 DAF to 24 DAF, followed by a slow enhancement from 24 DAF to 32 DAF (43.1% of DW), and then a stable increase up to the peak level (47.4%) from 32 DAF to 40 DAF (almost matured). Similarly, an accumulation pattern (a rapid increase from 24 DAF to 32 DAF) of ALA was also observed during perilla seed development, with a peak level of 70.19% of total FAs at 40 DAF. Collectively, the *PfGPAT9* expression pattern is in accordance with the dynamic accumulation of oil and ALA in developing seeds, providing further evidence for the potentially crucial role of *PfGPAT9* in seed oil synthesis and accumulation.

### 2.5. Isolation and Subcellular Localization of PfGPAT9 from P. frutescens

**Figure 5:**
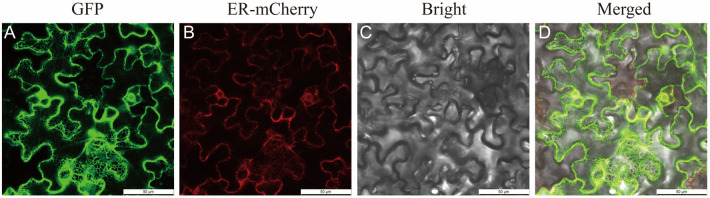
Subcellular localization of PfGPAT9 in *N. benthamiana* leaf epidermal cells. *Agrobacterium* GV3101 cells harboring either 35S::PfGPAT9::GFP or pCAMBIA1300-35S-ER-mCherry-HDEL were co-infiltrated into *N. benthamiana* leaf epidermal cells. The fluorescent signals were visualized under a laser scanning confocal microscope (Leica TCS SP8) at 36 h to 48 h after infiltrating. (**A**) Green fluorescent signals from 35S::PfGPAT9::GFP. (**B**) Red fluorescent signals from pCAMBIA1300-35S-ER-mCherry-HDEL. (**C**) Bright filed image. (**D**) Merged image of 35S::PfGPAT9::GFP with pCAMBIA1300-35S-ER-mCherry-HDEL. Bars = 50 µm.

The *PfGPAT9* gene was cloned via high-fidelity RT-PCR using gene-specific primers (Table S3), and the total cDNA templates were derived from developing seeds of the perilla variety ‘Jinzisu 1’ (Figure S2). The open reading frame (ORF) of *PfGPAT9* was 1116 bp and encoded a predicted protein consisting of 371 amino acids. To explore the subcellular localization of the PfGPAT9 protein, the ORF sequence of the *PfGPAT9* gene was inserted into the GFP fusion expression vector to construct the pCAMBIA1300/GFP vector. Then, the recombinant vector (35S::PfGPAT9::GFP) and the ER localization marker vector (pCAMBIA1300-35S-ER-mCherry-HDEL) were co-transformed into *N. benthamiana* leaves by *Agrobacterium*-mediated infiltration. As observed in Figure 5, the green fluorescent signals (GFP) from 35S::PfGPAT9::GFP were merged with the red fluorescent signals (mCherry) from pCAMBIA1300-35S-ER-mCherry-HDEL. Therefore, the PfGPAT9 protein was localized in the ER of the cells, consistent with the prediction based on the protein sequence.

### 2.6. Heterogenous Expression of PfGPAT9 Promotes Oil Accumulation in Yeast

In order to investigate the function of the *PfGPAT9* gene, we constructed a yeast expression vector pYES2.0 + *PfGPAT9* and introduced it into the wild-type (WT) strain INVSc1 of *Saccharomyces cerevisiae*. The empty vector pYES2.0 (EV) was used to transform the yeast as the negative control. The transgenic yeast cell lines harboring the *PfGPAT9* gene were obtained by PCR detection (Figure S3). Then, one independent line of *PfGPAT9*-transgenic with high expression was selected for phenotypic analysis, including examinations of lipid droplets, total oil content, and FA profiles.

**Figure 6:**
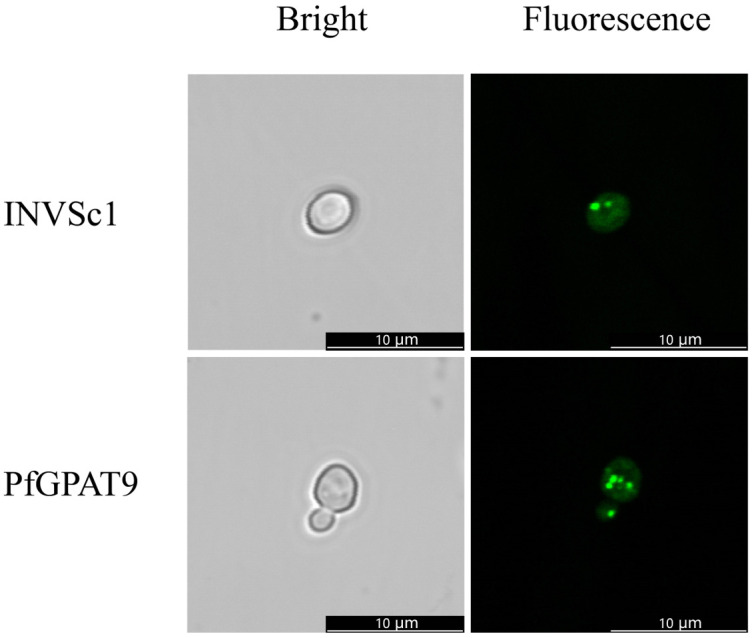
Lipid droplets in *PfGPAT9*-carrying yeast cells. The yeast cells were stained with BODIPY and examined by fluorescence microscopy. INVSc1 indicates the WT yeast cell stained with BODIPY. PfGPAT9 indicates the *PfGPAT9*-carrying yeast cell stained with BODIPY. Bars = 10 µm.

To explore the effect of heterologous expression of the *PfGPAT9* gene on lipid accumulation in the yeast, a positive transgenic yeast carrying the *PfGPAT9* gene was induced for expression. Firstly, the BODIPY fluorescent assay was employed to detect whether more lipid droplets were formed in the *PfGPAT9*-transgenic yeast line. As shown in Figure 6, the number of lipid droplets was increased in the *PfGPAT9*-transgenic yeast cells compared to the WT strain INVSc1.

**Figure 7:**
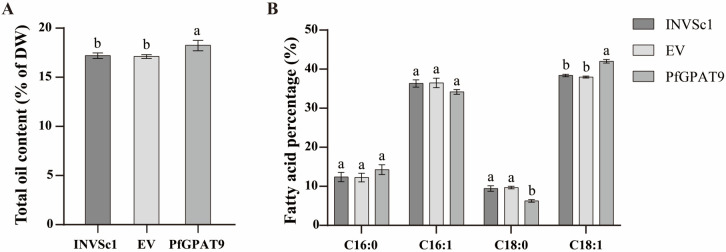
Total oil content (**A**) and fatty acid compositions (**B**) in yeast INVSc1 expressing the *PfGPAT9* gene. INVSc1 indicates the wild-type yeast strain; EV indicates the empty-vector pYES2.0-expressing yeast strain; and PfGPAT9 indicates the *PfGPAT9*-expressing yeast strain. Values are presented as mean ± SD (*n* = 3). Different lowercase letters indicate significant differences at *p* < 0.05.

Subsequently, the transgenic yeast cells, after induced expression, were freeze-dried under vacuum to determine the total oil content and fatty acid composition. As shown in Figure 7A, the *PfGPAT9*-carrying yeast accumulated a higher level of total oil (18.25% of DW) compared with the WT line (17.21% of DW) or the EV control (17.13% of DW). Furthermore, fatty acid methyl esters (FAME) from the WT, EV, and *PfGPAT9*-transgenic yeast strains were extracted, respectively, to determine the FA profiles using gas chromatography–mass spectrometry (GC–MS). All three yeast lines contained four FAs, including C16:0 (palmitic acid), C16:1 (palmitoleic acid), C18:0 (stearic acid), and C18:1 (oleic acid) (Figure 7B). The content of each FA component in the EV-carrying yeast was found to be comparable to that of the WT yeast. However, the contents of C18:1 and C16:0 in the *PfGPAT9*-carrying yeast line exhibited an increase of 1.88% and 3.64%, respectively, compared to WT control, accompanied by the decreases of C16:1 and C18:0 contents. Taken together, heterologous overexpression of the *PfGPAT9* gene from *P. frutescens* in yeast strain INVSc1 can promote lipid synthesis and accumulation, leading to an increase in total oil content and variation in the contents of some FA components.

### 2.7. PfGPAT9 Has Higher Substrate Preference towards Oleic Acid Than ALA

To test the substrate preference of enzymes encoded by *PfGPAT9*, the WT and *PfGPAT9*-carrying yeast lines were cultured with and without exogenous FA in the medium. The major FAs in the seed oil of *P. frutescens* are C18:3 (54–64%), C18:1 (14–23%), and C18:2 (11–16%) [61]. Subsequently, these three FAs (C18:3, C18:1, and C18:2) were supplemented into the medium, respectively. FA compositions in TAGs in these yeast cells were detected by GC–MS. As shown in Table 1, for the WT yeast cells, no significant change in FA compositions or their percentages was detected between the presence and absence of any of the three FAs in the medium. However, when cultured in the medium with each exogenous FA supply, the level of each FA was significantly different between the WT yeast and *PfGPAT9*-transgenic yeast. Remarkably, TAGs in the *PfGPAT9*-transgenic yeast line contained a significantly high level of C18:1 (70.54% of total FAs) and C18:3 (21.65% of total FAs) when cultured in the medium with the addition of C18:1 and C18:3, respectively, while C18:2 has no obvious change in the presence of exogenous C18:2. This UFA-feeding assay evidences that *PfGPAT9* has a substrate preference for UFAs, particularly for C18:1, followed by C18:3.

### 2.8. Heterogeneous Expression of PfGPAT9 Significantly Increases Contents of Total Oil and the Target FAs in Seeds and Non-Seed Tissues of Tobacco Plants

To further investigate the potentials of the *PfGPAT9* gene in plant genetic engineering to improve both oil yield and production of the desirable FAs (e.g., C18:1 and C18:3), plant constitutive overexpression vector pCAMBIA1303 (pCAMBIA1303 + *PfGPAT9*) and seed-specific expression vector pJC-Gly-DSRB (pJC-Gly-DSRB + *PfGPAT9*) were constructed (see Section 4) and then introduced into tobacco (*Nicotiana tobacum*) using *Agrobacterium*-mediated leaf disc transformation. At the same time, the empty vector pCAMBIA1303 or pJC-Gly-DSRB (EV) and untransformed (WT) tobacco plants were used as controls. The positive *PfGPAT9*-transgenic tobacco plant lines (homozygous T3 plants) were identified at both genomic and transcriptional levels by RT-PCR (Figure S4). One high-expressing transgenic tobacco line was selected for phenotypic analysis to examine total oil content, FA composition, and other agronomic traits.

**Figure 8:**
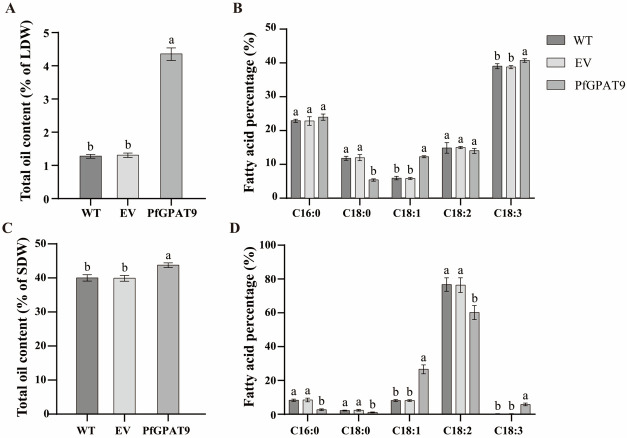
The total oil content and major fatty acid compositions in *PfGPAT9*-transgenic tobacco leaves (**A**,**B**) and seeds (**C**,**D**). (**A**,**B**) The total oil content (**A**) and major fatty acid compositions (**B**) in *PfGPAT9*-transgenic tobacco leaves. LDW indicates the dry weight of tobacco leaves. EV indicates the empty-vector pCAMBIA1303 transgenic tobacco plant lines. (**C**,**D**) The total oil content (**C**) and major fatty acid compositions (**D**) in *PfGPAT9*-transgenic tobacco seeds. SDW indicates the dry weight of tobacco seeds. EV indicates the seed-specific expression of empty-vector pJC-Gly-DSRB transgenic tobacco plant lines. WT indicates wild-type tobacco plant lines; PfGPAT9 indicates the *PfGPAT9*-transgenic tobacco plant lines. Values are presented as mean ± SD (*n* = 3). Different lowercase letters indicate significant differences at *p* < 0.05.

Total oil and fatty acid methyl esters (FAMEs) were extracted from the leaves and seeds of the transgenic homozygous tobacco lines. Then, the contents of the total oil and major FA components were measured (see Section 4). As shown in Figure 8, total oil contents in leaves and seeds exhibited no significant difference between the WT and EV, whereas the *PfGPAT9*-expressing tobacco lines showed a significant enhancement of total oil content by 2.82% and 3.74% in the leaves and seeds, respectively, compared to the controls (Figure 8A,C). Furthermore, no significant differences in FA profiles in leaves and seeds were detected between the WT and EV-transformed plant lines (Figure 8B,D). Compared to the WT lines, the *PfGPAT9*-expression tobacco line showed a significant enhancement in the content of C18:1 and C18:3 in leaf tissues, particularly with a 1.07-fold increase in C18:1, while the content of C18:0 was significantly reduced in the *PfGPAT9*-transgenic lines (Figure 8B). Similarly, FA profiles in tobacco seeds also exhibited such a change pattern between the *PfGPAT9*-expression and WT or EV lines, with a remarkable increase in 2.27-fold and 26.76-fold in C18:1 and C18:3 content, respectively, which were much higher than their counterparts in the WT lines (Figure 8D). In short, these data indicate that the heterogeneous PfGPAT9 protein can function efficiently in tobacco seeds and non-seed tissues to promote oil biosynthesis and exhibit a stronger substrate preference for C18:1 than C18:3.

### 2.9. Overexpression of PfGPAT9 Leads to No Significant Negative Effects on Other Agronomic Traits of Tobacco Plants

**Figure 9:**
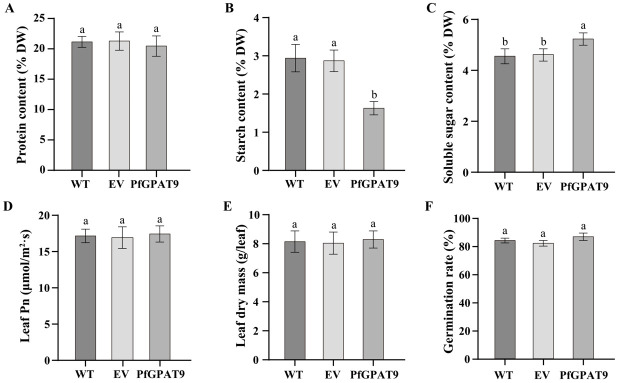
Other agronomic traits in the tobacco plants expressing the *PfGPAT9* gene. (**A**) Total protein content. (**B**) Total starch content. (**C**) Total soluble sugar content. (**D**) Leaf photosynthesis (Pn). (**E**) Leaf dry mass. (**F**) Seed germination rate. WT, wild-type tobacco plant; EV, the empty-vector pJC-Gly-DSRB transgenic tobacco plant; PfGPAT9, the *PfGPAT9*-transgenic tobacco plant. Values are presented as mean ± SD (*n* = 3). Different lowercase letters indicate significant differences at *p* < 0.05.

To evaluate whether *PfGPAT9* overexpression affects other agronomic traits in the heterogenous host, we further examined levels of protein, starch, and soluble sugar in mature seeds, leaf photosynthesis (Pn), dry mass, and seed germination rate of the *PfGPAT9*-transgenic tobacco lines. As shown in Figure 9A, the protein content in seeds was comparable between the control (WT and EV tobacco lines) and the transgenic plants overexpressing the *PfGPAT9* gene. Starch content decreased from 2.94% in the WT control to 1.63% in *PfGPAT9*-transgenic plants (Figure 9B). For soluble sugar content in seeds (Figure 9C), a small increase of 0.67% was detected in the *PfGPAT9*-transgenic plants compared to the controls. No obvious changes were detected for leaf photosynthesis (Figure 9D), leaf dry mass (Figure 9E), or seed germination rate (Figure 9F) among the samples tested. Taken together, these data demonstrate that heterogeneous overexpression of *PfGPAT9* in tobacco can significantly improve the carbon source into the lipid biosynthesis pathway, resulting in the enhancement of total oil and C18:1 accumulation without having a negative effect on leaf photosynthesis, plant growth, seed germination, or other agronomic traits. High-leaf and seed oils enriched with the desirable C18:1 are favorable for their utilization as high-quality oils in food and other industrial fields.

## 3. Discussion

Perilla is a distinctive oilseed crop renowned for its exceptional nutritional and medicinal properties. Particularly, perilla seed is one of the highest accumulators of ALA, being recognized as a desirable resource for human health [50]. However, limited knowledge is available for the molecular mechanism responsible for PUFA and storage lipids (oil) as well as other glycerolipids in this oil crop. GPAT catalyzes the initial and rate-limiting step of glycerolipid synthesis by transferring the acyl moiety to a glycerol-3-phosphate (G3P) molecule, thereby regulating the synthesis of TAG and other glycerolipids. Despite many studies in plants that have revealed the key role of GPATs in glycerolipid biosynthesis, perilla GPAT members and their functions remain unknown. Here, we present a genome-wide examination of the perilla PfGPAT family and, especially, the functional characterization of PfGPAT9 using ex vivo assay systems, including yeast and tobacco plant transformations, aiming to advance our understanding of GPAT functions in glycerolipid biosynthesis and different physiological processes and also to provide valuable candidate genes for plant lipid metabolic engineering.

### 3.1. Fourteen PfGPATs Classified to Three Distinct Groups May Function Diversely

Previously, genome-wide identification in various plants revealed multiple members of the GPAT family in plant genomes and different GPATs with different glycerolipid synthesizing abilities, such as ten GPATs in *Arabidopsis* [33], 20 GPATs in maize [38], and 85 GPATs in Gossypium (14, 16, 28, and 27 GPATs in *G. raimondii*, *G. arboreum*, *G. hirsutum*, and *G. barbadense*, respectively) [34]. This feature of GPAT was also identified in 39 species, including algae, basal plants (two mosses and one lycophyte), monocots, and eudicots [33]. For example, different members of the *Arabidopsis* AtGPAT family function in different subcellular apartments, with ATS1 (soluble form) in chloroplast, AtGPAT1-3 (membrane-bound form) in mitochondria, and AtGPAT4-9 (ER membrane-bound form) in ER, respectively [27,28,29,36,42].

In this study, a total of 14 PfGPAT members were identified in *P. frutescens* based on the published perilla genome database [51]. All these perilla PfGPATs were distinctly classified into three groups: Group I, Group II, and Group III (III-a, III-b, and III-c), as revealed by phylogenetic analysis (Figure 1), which was similar to those in *Arabidopsis* [28], maize [38], and other plants [33]. Each phylogenetic group contained at least one member from both the perilla and the model plant *Arabidopsis*. All these PfGPATs were detected to contain the four highly conserved motifs (Blocks I–IV). Blocks I, III, and IV contain amino acid residues essential for GPAT catalysis (His-H and Asp-D in Block I, Gly-G in Block III, and Pro-P in Block IV), while Blocks II and III contain amino acid residues essential for binding G3P (Arg-R in Block II, and Glu-E in Block III) [44]. However, distinct variations in the conserved motifs were identified in different groups of PfGPATs (Figure 2), indicating that functional divergence may exist for different PfGPAT members. Furthermore, the analysis of MEME motifs and gene structure of *PfGPATs* (Figure 3) showed that *PfGPAT* members in the same group contained the same conserved motifs and intron/exon structures but varied between different groups. *GPAT* genes within the same groups showed similar intron/exon structures, indicating that these genes within the same group might have diverged from a common ancestor.

Gene expression patterns can provide valuable clues for understanding gene function [62]. Previous studies reported that GPAT members presented variable gene expression patterns in various tissues/organs or under different stress conditions [33], suggesting that they may be differentially regulated depending on plant tissues. For example, a number of cotton *GhGPAT* genes were upregulated in roots under salt stress, but only a few upregulated *GhGPAT* genes were found in leaves compared with roots [34]. Moreover, soluble *GPAT* genes were detected to be predominantly expressed in green tissues and involved in chloroplast lipid biosynthesis in *A. thaliana*, *G. max,* and *Z. mays* [33]. A gene encoding a soluble GPAT of *Helianthus annuus* (*HaPLSB*) [63] also showed a similar expression pattern, with an increased expression during cotyledon development, which was consistent with the elevated rate of de novo chloroplast membrane lipid biosynthesis in the target tissue. Our results showed that all 14 *PfGPAT* genes were expressed during perilla seed development, but their expression levels varied greatly (Figure 4A), with other *PfGPATs* having an opposite temporal pattern from *PfGPAT9*. These expression data suggest that these genes may perform diverse functions in perilla, although their dynamics of expression profiles in various tissues/organs and their detailed functions require further explored in multiple dimensions.

### 3.2. ER-Localized PfGPAT9 Functions Crucially in TAG Assembly, with Substrate Preference to UFAs (OA > ALA)

Our phylogenetic analysis revealed that PfGPAT9 was closely related to AtGPAT9 (Figure 2). Like AtGPAT9 [28] and other plants such as *P. patens* GPAT9 [44], PfGPAT9 is also localized in ER (Figure 6), where TAG is synthesized and an oil body is formed. Moreover, the *PfGPAT9* gene exhibited a high expression level in perilla seeds, displaying a temporal pattern of initial upregulation followed by downregulation with seed development (Figure 4B). Such expression features of the *PfGPAT9* gene in seeds were also similar to those of the *AtGPAT9* gene in *Arabidopsis* seeds [43] and *RcGPAT9* in castor, where *RcGPAT9* presents higher expression compared to other GPATs in the endosperm tissue of seed [64], implying that PfGPAT9 may function significantly in seed oil accumulation similarly to AtGPAT9 and RcGPAT9.

To investigate this function of PfPGAT9, an association analysis was conducted between the dynamics of total oil accumulation and the *PfPGAT9* gene expression pattern during perilla seed development (Figure 4B,C). Significantly, *PfGPAT9* expression dynamics (Figure 4B) were highly correlated with total oil and ALA accumulation patterns during perilla seed development (Figure 4C), showing again that PfGPAT9 may play a key role in total oil or ALA biosynthesis and accumulation in perilla seeds.

Since the genetic transformation of perilla was not established yet, we employed an ex vivo enzymatic assay based on a yeast expression system to functionally characterize PfGPAT9. Heterogeneous overexpression of *PfGPAT9* resulted in a great enhancement of lipid droplets (Figure 6) and total oil content (Figure 7A) in the transgenic yeast INVSc1. Meanwhile, the OA (C18:1) content was significantly increased in the *PfGPAT9*-transgenic yeast, whereas the SA (C18:0) content was reduced accordingly (Figure 7B). Thus, the PfGPAT9 enzyme is an active acyltransferase functioning crucially in TAG biosynthesis and possible substrate preference to OA. More importantly, our feeding assay with three UFAs (C18:3, C18:2, and C18:1, the most abundant FAs in perilla seed oil) demonstrated that a higher level of oil production was obtained in the *PfGPAT9*-transgenic yeast cultured in medium supplemented with exogenous C18:1, followed by adding exogenous ALA (C18:3) (Table 1). These data evidence that PfGPAT9 has a stronger substrate specificity for C18:1 than C18:3, but no selection for C18:2. Such enzyme features of PfGPAT9 combined with its expression profile during seed development clearly demonstrate that PfGPAT9 is one of the main contributors to the high level of total oil and UFA (C18:1 and C18:3) accumulation in perilla seeds, despite the need for future examinations in vivo.

In accordance with our finding that PfGPAT9 is a key player for oil biosynthesis, GPAT9 from other plants was also detected to be involved in TAG biosynthesis, even in several algal species producing an abundance of TAGs [47,65], including the model unicellular green alga *Chlamydomonas reinhardtii* [66]. Moreover, different substrate specificities were found for GPAT9s from various plant species. For example, AtGPAT9 also showed the highest substrate preference for C18:1-CoA relative to other FA-CoAs (e.g., C16:0-CoA, C18:0-CoA, and C18:3-CoA) [67]. But sunflower HaGPAT9-1 has the strongest substrate preference for C16:0-CoA, followed by C18:2-CoA and C18:1-CoA [32]. Additionally, 16:0-CoA was also the favorite substrate of a GPAT9 homology (CrGPATer, localized in ER) from *Chlamydomonas reinhardtii*, followed by 18:1-CoA [66]. Possibly, such GPAT9 preferences for FAs with different saturation degrees represent a mechanism to maintain the homeostasis between different UFAs in the target tissue/organ. As a single-copy member in most organisms, the GPAT9 mutant displayed the lethal-embryo phenotype in *Arabidopsis* [42] and the lower plant *P. patens* [44], indicating that GPAT9 plays an essential role for plants in addition to its function in glycerolipid synthesis. Nevertheless, the mechanism responsible for GPAT9 roles and their differences derived from diverse plants need further investigation.

It is worth noting that other acyltransferases besides GPATs were reported to have substrate specificity for different acyl-CoAs. For example, enzymatic assays of RcLPAT2 from castor (*Ricinus communis*) showed a preference for ricinoleoyl-CoA over other fatty acid thioesters when ricinoleoyl-LPA is used as the acyl acceptor, while oleoyl-CoA is the preferred substrate when oleoyl-LPA is employed [68]. However, RcLPATB displayed a broad specificity for the acyl-CoAs, with saturated fatty acids (C12:0–C16:0) being the preferred substrates. Moreover, RcDGAT2, RcPDAT1-2, and RcPDAT1A also exhibited substrate preference for ricinoleic acid, an unusual fatty acid [69,70,71]. Therefore, the synergistic reaction of these acyltransferase isoenzymes controls the efficient channeling of ricinoleic acid into castor bean triacylglycerol. Possibly, the levels of different FAs incorporated into TAGs in perilla seed oil are determined by multiple acyltransferase members showing substrate selectivity for different FAs (e.g., C16:0, C18:0, C18:1, C18:2, and C18:3), which need future investigations in detail.

### 3.3. PfGPAT9 Is a Desirable Target in Metabolic Engineering to Increase Production of Storage Oils Enriched with Valuable UFAs in Plant Seed and Non-Seed Tissues

As the key rate-committed enzyme for the first acylation reaction in TAG biosynthesis, GPAT was reported to be a suitable target in biotechnology to improve vegetable oil yield and quality. For example, heterologous expression of the plastid-type *GPAT* gene of safflower in *Arabidopsis* led to a significant increase (10% to 21%) in seed oil content compared to the wild-type plant [72]. Overexpression of the tomato *GPAT* gene in *Arabidopsis* increased the UFA content within thylakoid membranes and alleviated salt damage in *Arabidopsis* [30]. In addition, two *GPAT* genes from *Jatropha curcas* were overexpressed in Arabidopsis, leading to an improvement in the total oil content of transgenic plants [73]. Overexpression of the *PtGPAT* gene in the diatom *Phaeodactylum tricornutum* significantly increased the accumulation of UFA and total oil in the transgenic lines [74].

Of the GPATs identified so far, GAPT9 was recently used as the genetic modification target in lipid metabolic engineering. For instance, overexpression and downregulation of *AtGPAT9* in *Arabidopsis* resulted in changes in seed size as well as seed oil content and composition [43]. Genetic modification of *Arabidopsis* AtGPAT9 also enhanced the production of both polar and non-polar lipids in leaves, as well as lipid droplets in pollen [43]. The ectopic expression of the *PpGPAT9* gene from *Physcomitrella patens* increased the size of the seeds and embryonic cells, leading to higher levels of C18:3 and C20:2 in the transgenic *Arabidopsis*, despite the fact that C20:2 is not a major component in wild-type *Arabidopsis* seeds [44]. Overexpression of *CmGPAT9* from *C. merolae* also resulted in increased oil accumulation in the host cells [46]. Heterologous expression of a *GPAT9* homolog from an oleaginous microalga, *Lobosphaera incisa,* increased TAG content by 50% or more in the transgenic *C. reinhardtii* [47]. Overexpression of *CrGPATer* (*GPAT9*) from *C. reinhardtii* in yeast (*S. cerevisiae*) significantly increased the content of TAGs and sterol esters in the transgenic yeast cells [66].

In this study, we employed the model plant tobacco as the host to overexpress the *PfGPAT9* gene identified here to evaluate its potential in engineering to improve oil yield and FA compositions in both seed and non-seed tissues. Our results showed that the oil content in leaves and seeds of transgenic tobacco lines overexpressing *PfGPAT9* was 2.1-fold (Figure 8A) and 0.09-fold (Figure 8C) higher than that of the wild type, respectively. Furthermore, the leafy oil within PfGPAT9-transgenic tobacco exhibited 6.33% and 1.73% increases in C18:1 and C18:3 contents compared to WT plants, respectively, accompanied by a substantial reduction in C18:0 (Figure 8B). The seed oil of *PfGPAT9*-transgenic tobacco also showed 18.45% and 5.62% increases in C18:1 and C18:3 contents compared to WT lines, respectively, along with notable reductions in C16:0, C18:0, and C18:2 levels (Figure 8D). Remarkably, C18:3 is not the major FA component in tobacco seeds. The significant increases in C18:1 and C18:3 levels in the transgenic leaves and seeds again proved the substrate specificity of PfGPAT9 to C18:1 and C18:3. Of particular importance, the *PfGPAT9*-transgenic tobacco plants showed no negative effects on other agronomic traits, including plant growth and seed germination rate, as well as other morphological and developmental features (Figure 9).

Taken together, all these indicate GPAT9 as a promising target that can be used for the increased production of storage oils in transgenic oilseed plants, despite different GPAT9s from diverse plant species exhibiting differential impacts on TAG-associated FA profiles. As described above, overexpression of *PfGPAT9* increased the content of oils enriched with UFAs (C18:1 > C18:3) in the host. Such oil with a suitable ratio of C18:1 and C18:3 exhibits favorable nutritional and commercial values. The vegetable oils with higher levels of C18:1 have high oxidative stability, making them suitable for human health and the food industry, while the oils with higher levels of C18:3 have high oxidation instability but are health-promoting, with anti-oxidation as a key issue during the production of 18:3-based commodities. Particularly, our study highlights that PfGPAT9 from perilla is a novel genetic resource in lipid metabolic engineering for enhancing storage oil rich in valuable UFAs (e.g., C18:1 and C18:3) in oleaginous microbes and plants, thereby promoting the sustainable production of designed oils or high-quality vegetable oils in the world.

Based on our current findings showing the crucial function of PfGPAT9 in the biosynthesis of 18:1/18:3-rich TAGs, future investigations should focus on the regulatory mechanisms of PfGPAT9 or its homolog in perilla and other oilseed crops. Particularly, knockdown/knockout and overexpression of the *PfGPAT* members should be performed in the perilla to precisely characterize their functions following the establishment of the perilla genetic transformation system. Moreover, the substrate specificity of other acyltransferases, such as LPATs, DGATs, and PDATs, needs to be examined in perilla. In fact, our ongoing work indicates that PfLPAT and PfDGAT have different substrate preferences for C18:1, C18:2, and C18:3, respectively (unpublished data). Integrating all these data from these investigations will uncover the exact mechanism of biosynthesis and regulation of perilla seed oils enriched with UFAs, benefiting the genetic improvement of oil yield and quality in other oilseeds.

## 4. Materials and Methods

### 4.1. Plant Materials and Growth Conditions

*Perilla frutescens* (variety ‘Jinzisu 1’) was cultivated at the agricultural station of Shanxi Agricultural University (112°58′ E, 37°42′ N), Shanxi Province, China. The roots, stems, and leaves of the perilla were collected at the six-leaf period of seedling growth, and flowers were collected at the flowering period. Seeds of perilla were collected at 8 DAF (days after flowering), 16 DAF, 24 DAF, 32 DAF, and 40 DAF. All tissues were immediately frozen in liquid nitrogen and stored at −80 °C for further use.

The tobacco *Nicotiana benthamiana* was selected for the analysis of the subcellular localization of the PfGPAT9 protein. The common tobacco *Nicotiana tabacum* (*Sumsun* NN, SNN) was used for the heterologous overexpression of the *PfGPAT9* gene. All tobacco plants were cultivated under controlled conditions in a climate-controlled chamber, maintaining a light/dark photoperiod of 16 h/8 h at a temperature of 25 °C.

### 4.2. Identification and Sequence Characterization of GPAT Genes from P. frutescens

#### 4.2.1. Identification of *GPAT* Genes in the *P. frutescens* Genome and Chromosomal Localization

*P. frutescens* genome annotation files were downloaded from *Perilla frutescens* var. *frutescens* (https://www.ncbi.nlm.nih.gov/genome/?term=txid48386 (accessed on 6 January 2023)), and *AtGPAT* sequences from *Arabidopsis* were downloaded from The Arabidopsis Information Resource (TAIR) (https://www.arabidopsis.org/index.jsp (accessed on 6 January 2023)). Two different methods were used to identify the *GPAT* genes in *P. frutescens*. Firstly, using AtGPAT protein sequences as a probe, the BLAST tool of BioEdit was used to identify candidate PfGPATs, with the E value cut-off set at 0.001. Then, each candidate PfGPAT was further examined to confirm the existence of conserved domains by the NCBI-CDD (https://www.ncbi.nlm.nih.gov/Structure/cdd/wrpsb.cgi (accessed on 7 January 2023)). The online bioinformatics tool ProtParam (https://web.expasy.org/protparam/ (accessed on 7 January 2023)) was used to predict the basic physical and chemical properties of the PfGPAT proteins. The PSORTII (http://psort.hgc.jp/form2.html (accessed on 7 January 2023)) was used to predict the subcellular localization of the PfGPAT proteins. The online tool TMHMM Server ver.2.0 (http://www.cbs.dtu.dk/services/TMHMM-2.0/ (accessed on 7 January 2023)) was used to analyze the transmembrane domains of PfGPAT proteins.

The chromosomal positions of all the *PfGPAT* genes were extracted from the genome annotation file. Finally, the physical position of each *PfGPAT* gene on the chromosome was mapped from the short-arm to long-arm telomeres using MapChart 2.32 [75].

#### 4.2.2. Multiple Sequence Alignment and Phylogenetic Analysis

A multiple sequence alignment of the PfGPAT proteins was performed by the Jalview 2.11.2.7 software. Based on the alignment of the GPAT conserved domains of PfGPATs and AtGPATs, a phylogenetic tree was constructed with MEGA11 using the neighbor-joining (N-J) method with 1000 bootstrap replicates. All the identified PfGPAT sequences were classified into distinct groups according to the classification of AtGPAT sequences.

#### 4.2.3. Gene Structure and Conserved Motif Analysis

The full-length (from a genomic file) and CDS sequences of the *PfGPAT* genes were submitted to the online tool Gene Structure Display Server (GSDS, http://gsds.gao-lab.org/index.php (accessed on 7 January 2023)) to analyze the gene structure of the PfGPAT members. Conserved motif analysis was performed using the online tool MEME (http://meme-suite.org/tools/meme (accessed on 7 January 2023)). Parameters were set as follows: the maximum number of motifs was set to 6, and the motif width range was set from 6 to 50. A total of six conserved motifs were identified, and the results were embellished using TBtools 2.0.

### 4.3. Expression Analysis of PfGPAT Genes in Different Tissues and Correlation with Oil Synthesis

#### 4.3.1. Expression Profiles Analysis of *PfGPAT* Genes Based on RNA-Sequencing Data

To investigate the profiles in expression of the *PfGPAT* gene across different stages of seeds, we acquired perilla transcriptome data of all 14 *PfGPAT* genes from our house transcriptome sequencing database (performed by Genedenovo Biotechnology Co., Ltd., Guangzhou, China). The expression levels of *PfGPAT* genes were quantified on the basis of their fragments per kilobase of exon per million fragments mapped (FPKM) reads. The log values of the FPKMs were used to construct the heatmap by TBtools.

#### 4.3.2. Total RNA Extraction and RT-qPCR Analysis

Total RNA was extracted using the EASY spin Plus Plant RNA kit (Aidlab Biotechnologies Co., Ltd., Beijing, China). The isolated pure RNA was reversed to the first-strand cDNAs using the StarScript II RT Mix with gDNA (GenStar BioSolutions, Beijing, China). RT-qPCR primers were designed using Primer6 and are listed in Table S2.

RT-qPCR was performed using the TB Green^®^ Premix Ex Taq^™^ II (Tli RNaseH Plus) (TaKaRa, Beijing, China) on a CFX96PCR system (Bio-Rad, Hercules, CA, USA). The RT-qPCR reaction conditions were one cycle of pre-denaturation at 95 °C for 2 min; 40 cycles of 95 °C for 30 s, 60 °C for 30 s, and 72 °C for 30 s; dissolution at 95 °C for 5 s; and 60 °C for 1 min. PfActin was used as the internal reference gene. All the experiments were repeated three times, and the relative expression levels were calculated by the 2^−ΔΔCt^ method [76].

#### 4.3.3. Total Oil Extraction and Fatty Acid Analysis

Freeze-dried perilla seeds were ground into powder, and 0.05 g powder was used for total oil extraction using the chloroform-methanol method described by El Tahchy et al. [77]. The total oil content was obtained by weighing the change in weight of the glass tube. The FA methyl esters (FAMEs) were prepared according to the method described by Zhang et al. [78]. FAMEs were analyzed using an Agilent 7890B gas chromatograph equipped with a hydrogen flame detector and an HP-88 column (100 m × 0.25 mm × 0.20 μm). The inlet and oven start temperatures were set to 250 °C and 140 °C, respectively. High-purity nitrogen was used as a carrier gas. Methyl heptadecanoate (C17:0) was used as an internal standard, and a standard curve method was used as the quantitative approach to identify FAMEs [6]. Each sample was analyzed in triplicate.

### 4.4. PfGPAT9 Gene Cloning and Vectors Construction

We selected the ORF sequence of the *PfGPAT9* gene as the template to design gene-specific primers with *Apa* I (5′-end) and *Pst* I (3′-end) sites (primers listed in Table S3). The RT-PCR amplification was performed using the *TransStart^®^ FastPfu* Fly DNA Polymerase (TransGen Biotech, Beijing, China). The RT-PCR product of the *PfGPAT9* gene was ligated into the pCAMBIA1303 expression vector (pCAMBIA1303 + *PfGPAT9*, Figure S5) using the ClonExpress Ultra One Step Cloning Kit (Vazyme) and then transformed into *Escherichia coli* DH5α. The positive clones were confirmed through PCR detection and gene sequencing (Tsingke Biotechnology Co., Ltd., Xi’an, China).

The ORF of *PfGPAT9* was amplified from the recombinant vector pCAMBIA1303 + *PfGPAT9* using gene-specific primers with *Xba* I (5′-end) and *Pst* I (3′-end) sites (primers listed in Table S3). *PfGPAT9* ORF was then inserted into the pCAMBIA1300-GFP vector, resulting in the fusion expression vector pCAMBIA1300 + *PfGPAT9*/GFP (Figure S5).

The ORF of *PfGPAT9* was amplified from the recombinant vector pCAMBIA1303 + *PfGPAT9* using gene-specific primers with *Sac* I (5′-end) and *Xba* I (3′-end) sites (primers listed in Table S3). *PfGPAT9* ORF was then inserted into the yeast expression vector pYES2.0 under the control of the inducible promoter GAL1, and a recombinant yeast expression vector pYES2.0 + *PfGPAT9* was obtained (Figure S5).

The ORF of *PfGPAT9* was amplified from the recombinant vector pCAMBIA1303 + *PfGPAT9* using gene-specific primers with *Sac* I (5′-end) and *Xba* I (3′-end) sites (primers listed in Table S3) and then inserted into the seed-specific expression vector pJC-Gly-DSRB (pJC-Gly-DSRB + *PfGPAT9*) (Figure S5).

Recombinant vector pCAMBIA1300 + *PfGPAT9*/GFP was used to analyze the subcellular localization of the PfGPAT9 protein. pYES2.0 + *PfGPAT9* was used for the functional analysis of *PfGPAT9* gene overexpression in yeast. pCAMBIA1303 + *PfGPAT9* and pJC-Gly-DSRB + *PfGPAT9* were used for functional *PfGPAT9* gene heterologous overexpression in tobacco.

### 4.5. Subcellular Localization Analysis of PfGPAT9 Protein

The recombinant fused vector pCAMBIA1300 + *PfGPAT9*/GFP and the ER localization marker vector pCAMBIA1300-35S-ER-mCherry-HDEL were transformed into *Agrobacterium* GV3101 through the liquid nitrogen freeze–thaw method, respectively, and then transiently co-expressed in *N. benthamiana* leaf epidermal cells by *Agrobacterium* infiltration. The transfected tobacco plants were cultured at 25 °C (with a 16 h/8 h light/darkness photoperiod) for 36 h to 48 h and subsequently observed under a laser scanning confocal microscope (Leica TCS SP8). The cells containing GFP fluorescence were excited at 488 nm, and emissions were recorded at 510–550 nm, while mCherry was excited at 587 nm, and emissions were recorded at 610–650 nm.

### 4.6. Overexpression of PfGPAT9 in the S. cerevisiae INVSc1

To explore the functional GPAT activity of the PfGPAT9 protein, the recombinant plasmid pYES2.0 + *PfGPAT9* was transformed into INVSc1-competent cells of *S. cerevisiae* using the Yeast Transformation Kit (Coolaber Technology Company, Beijing, China). Also, the empty vector (EV) pYES2.0 was transformed into the INVSc1 strain as the negative control. Correct transformants were then confirmed by growth on synthetic 2% (*w*/*v*) glucose medium lacking uracil (SC-Ura, Coolaber Technology Company, Beijing, China) and RT-PCR assays (primers listed in Table S3).

After induction with galactose for 2 days, *PfGPAT9*-transgenic INVSc1 was resuspended to an optical density (OD_600_) of approximately 0.2. Yeast cell suspensions were mixed with equal volumes of BODIPY 505/515 working solution and stained for 10 to 30 min in the dark. Subsequently, the quantity and size of lipid droplets in yeast cells were observed under a laser scanning confocal microscope (Leica TCS SP8).

To examine the FA substrate preferences of PfGPAT9, we supplemented C18:1 (oleic acid, OA), C18:2 (linoleic acid, LA), and C18:3 (α-linolenic acid, ALA) into the medium with a final concentration of each fatty acid of 1 mmol/L [6].

All the yeast cells, after culturing, were enriched by centrifugation, vacuum-dried, and subjected to lipid extraction and analysis by the methods described previously (see Section 4.3.3).

### 4.7. Heterologous Overexpression of PfGPAT9 in Tobacco Plant

The recombinant plant overexpression vector pCAMBIA1303 + *PfGPAT9* was transformed into *Agrobacterium* GV3101 through the liquid nitrogen freeze–thaw method and then heterologously overexpressed in leaf discs of 30-day N. tabacum sterile tobacco seedlings by the *Agrobacterium*-mediated method [6]. Transgenic shoots were selected on Hygromycin B (5 mg/mL) and were rooted in 1/2 MS medium containing Hygromycin B (5 mg/mL). Simultaneously, RT-PCR was used to detect the effective expression of the *PfGPAT9* gene (primers listed in Table S3). Finally, the homozygous tobacco plants with robust growth were transplanted into the nursery substrate and then collected for phenotypic analysis.

Lipid extraction and analysis of *PfGPAT9*-transgenic tobacco were conducted using the methods described previously (see Section 4.3.3). The protein, starch, and soluble sugar were extracted and measured using the BCA Protein Assay Kit, Starch Content Assay Kit, and Plant Soluble Sugar Content Assay Kit (Beijing Solarbio Science & Technology Co., Ltd., Beijing, China), respectively. Meanwhile, the leaf photosynthetic rate (Pn), leaf dry mass, and seed germination rate of transgenic tobacco plant lines were analyzed.

### 4.8. Statistical Analysis

All experiments were biologically repeated three times. The experimental data were analyzed using the single-factor multiple comparison method in DPS software version 9.01. Values are presented as mean ± SD (*n* = 3). Significant differences between treatments were labeled as * at *p* < 0.05 or ** at *p* < 0.01.

## 5. Conclusions

In this study, a total of 14 PfGPAT members were identified from the *P. frutescens* genome and classified into three distinct groups (Groups I, II, and III) based on phylogenetic, gene structure, and protein property analysis. ER-localized PfGPAT9 exhibited the gene expression pattern consistent with the dynamics of total oil and ALA accumulations during the seed development of the perilla. Functional characterization of PfGPAT9 using ex vivo assay systems, including yeast and tobacco plant transformations, revealed that PfGPAT9 had strong GPAT enzymatic activity and a high substrate preference for OA (18:1), followed by ALA (18:3), which functions crucially in TAG biosynthesis. Our findings provide a solid foundation for further understanding the diverse functions of PfGPAT family members, demonstrating PfGPAT9 as a novel genetic engineering target for improving oil yield and quality in oilseed crops, thereby promoting sustainable production of high-value vegetable oils.

## Figures and Tables

**Table 1:** Fatty acid compositions in total fatty acids of *PfGPAT9*-expressing yeast cultured with the feeding of exogenous UFA.

Strains		Fatty Acid Composition (% of Total FAs)					
		C16:0	C16:1	C18:0	C18:1	C18:2	C18:3
Unfed	INVSc1	12.38 ± 0.99	36.34 ± 1.52	9.44 ± 0.70	38.38 ± 1.94		
	PfGPAT9	14.26 ± 1.95	34.18 ± 1.18	6.27 ± 0.63 **	42.02 ± 1.74		
C18:1-fed	INVSc1	11.45 ± 0.98	35.16 ± 1.85	8.22 ± 0.82	41.98 ± 2.11		
	PfGPAT9	6.23 ± 0.88 **	16.52 ± 1.18 **	3.46 ± 0.44 **	70.54 ± 2.57 **		
C18:2-fed	INVSc1	12.01 ± 1.23	32.85 ± 1.70	10.49 ± 0.97	32.31 ± 1.67	9.16 ± 0.90	
	PfGPAT9	7.96 ± 0.94 **	31.28 ± 1.73 **	8.34 ± 0.75	36.09 ± 2.29	13.21 ± 1.16 **	
C18:3-fed	INVSc1	12.46 ± 1.21	34.19 ± 2.27	8.98 ± 0.42	35.15 ± 1.87		6.13 ± 0.33
	PfGPAT9	10.63 ± 0.70	29.14 ± 1.12 **	6.27 ± 0.80 **	29.28 ± 0.96 **		21.65 ± 1.64 **

Unfed indicates that INVSc1 and *PfGPAT9*-transgenic yeast strains were cultured without the presence of exogenous FA in the medium. C18:1-fed, C18:2-fed, and C18:3-fed indicate that INVSc1 and *PfGPAT9*-transgenic yeast strains were cultured in the presence of exogenous FA (C18:1, C18:2, and C18:3) in the medium. Each FA composition was measured as a percentage of total FAs. Values are presented as mean ± SD (*n* = 3). ** *p* < 0.01.

## Data Availability

Data supporting the discovery of our work are available within the paper and its Supplementary Materials.

## Supplementary Materials

The supporting information can be downloaded at: https://www.mdpi.com/article/10.3390/ijms242015106/s1.
