# Supplementary material for: Genome-Wide Analysis of Glycerol-3-Phosphate Acyltransferase (GPAT) Family in Perilla frutescens and Functional Characterization of PfGPAT9 Crucial for Biosynthesis of Storage Oils Rich in High-Value Lipids
Source: Int J Mol Sci. 2023 Oct 12;24(20):15106. doi: 10.3390/ijms242015106 (PMC10606570; doi:10.3390/ijms242015106)
Supplement: Supplementary file 1 [file ijms-24-15106-s001.zip › ijms-2624199-supplementary.pdf]

Table S1. GPAT protein IDs of *P. frutescens* and *Arabidopsis*.

**Protein**	**Protein** ID	**Protein**	**Protein** ID
PfATS1-1	KAH6788306.1	AtGPAT1	NP_563768.1
PfATS1-2	KAH6771592.1	AtGPAT2	NP_563651.1
PfGPAT9	KAH6811989.1	AtGPAT3	NP_192104.1
PfGPAT1	KAH6785423.1	AtGPAT4	NP_171667.1
PfGPAT5-1	KAH6777675.1	AtGPAT5	NP_187750.1
PfGPAT5-2	KAH6769216.1	AtGPAT6	NP_181346.1
PfGPAT7-1	KAH6805671.1	AtGPAT7	NP_196227.1
PfGPAT7-2	KAH6802360.1	AtGPAT8	NP_191950.2
PfGPAT4	KAH6813183.1	AtGPAT9	NP_568925.1
PfGPAT6-1	KAH6777730.1	AtATS1	NP_174499.1
PfGPAT6-2	KAH6769243.1		
PfGPAT6-3	KAH6806876.1		
PfGPAT6-4	KAH6798376.1		
PfGPAT8	KAH6812091.1		

Table S2. Basic physicochemical properties of PfGPAT proteins.

Group	Gene	Gene ID	Chr	CDS (bp)	Protein (aa)	MW (kDa)	pI	GRAVY	Instability index	Predicted subcellular localization	Predicted transmembrane domains
I	PfATS1-1	C2S51_000050	02	1365	454	50.36	6.14	−0.209	49.79	cp	0
I	PfATS1-2	C2S51_000133	05	1377	458	50.76	5.81	−0.217	50.23	cp	0
II	PfGPAT9	C2S51_025751	12	1116	371	42.89	8.96	−0.102	47.51	ER	3
III-a	PfGPAT1	C2S51_037878	20	1248	415	46.11	8.94	0.13	35.07	mt	2
III-b	PfGPAT5-1	C2S51_008987	04	1491	496	54.72	9.07	0.247	36.25	ER	2
III-b	PfGPAT5-2	C2S51_014552	06	1491	496	54.59	8.99	0.245	35.21	ER	2
III-b	PfGPAT7-1	C2S51_030502	15	1422	473	52.68	9.57	0.224	39.49	ER	2
III-b	PfGPAT7-2	C2S51_033806	17	1482	493	55.03	9.44	0.206	37.24	ER	2
III-c	PfGPAT4	C2S51_022201	11	1506	501	55.53	9.2	0.155	36.44	mt	3
III-c	PfGPAT6-1	C2S51_009042	04	1506	501	56.11	9.22	0.06	37.21	ER	2
III-c	PfGPAT6-2	C2S51_014579	06	1506	501	56.10	9.32	0.062	37.02	ER	2
III-c	PfGPAT6-3	C2S51_027984	14	1509	502	56.75	9.39	−0.007	40.78	ER	2
III-c	PfGPAT6-4	C2S51_034860	18	1509	502	56.75	9.49	0.009	40.6	ER	2
III-c	PfGPAT8	C2S51_025853	12	1506	501	55.57	9.25	0.144	36.3	mt	3

ER, endoplasmic reticulum; cp, chloroplast; mt, mitochondrion.

Table S3. The primers used in this study.

Primer names	Forward primer (5′→3′)	Reverse primer (5′→3′)
PfGPAT9-q	TTCGCCAACTCTGACTGA	CAACTCCACAAGACCTCTC
PfGPAT9-pCAMBIA1300	ATACACCAAATCGACTCTAGAATGAGT AAGCTCAACACATCGAGC	GTCGACCCCGGGCCCCTGCAGTTTCTCT TCCAGGCGACGG
35S-GFP	GCCTTGCTTCCTATTATATCTTCCCAAAT TACC	GAACTTGTGGCCGTTTACGTCGC
PfGPAT9-pYES2.0	ATTAAGCTTGGTACCGAGCTCATGAGTA AGCTCAACACATCGAGC	TACATGATGCGGCCCTCTAGACTATTTC TCTTCCAGGCGACGG
T7/CYC1	TAATACGACTCACTATAGGG	GTGACATAACTAATTACATGATG
PfGPAT9-pCAMBIA1303	TCGGCATGCAGCGCTGGGCCCATGAGT AAGCTCAACACATCGAGC	TCCTCTAGAGTCGACCTGCAGCTATTTC TCTTCCAGGCGACGG
M13	GTAAAACGACGGCCAGT	CAGGAAACAGCTATGAC
PfGPAT9-DSRB	CACCATGTTGGGCCCGGCGCGCCATGA GTAAGCTCAACACATCGAGC	GTTGTCACATACATCGGCGCGCCCTATT TCTCTTCCAGGCGACGG

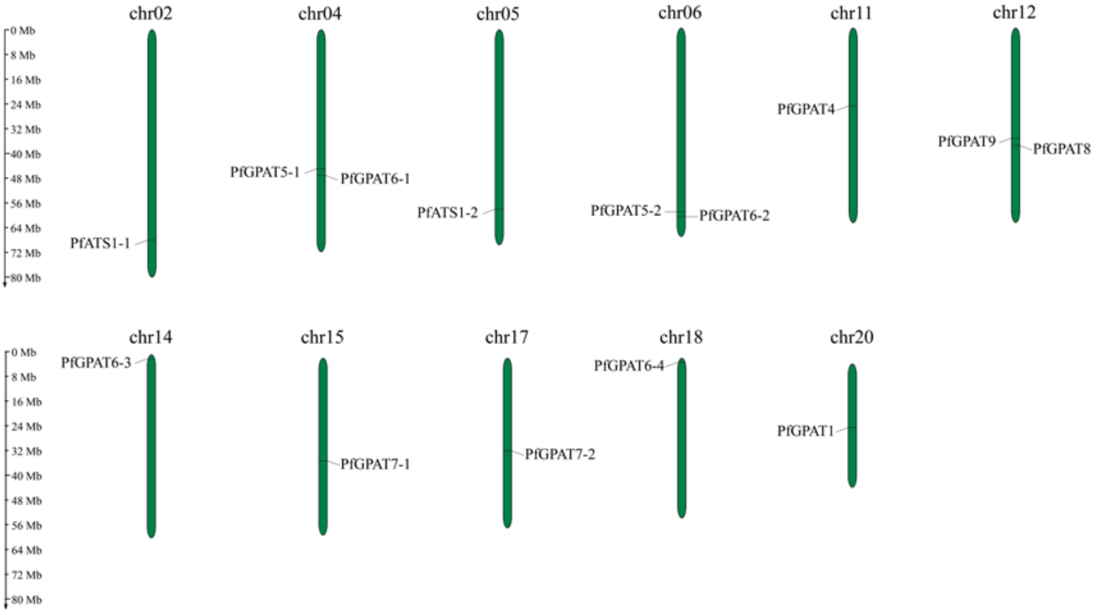

Figure S1. Chromosomal locations of PfGPAT genes. The relative length of the chromosomes is millions of base pairs (Mb).

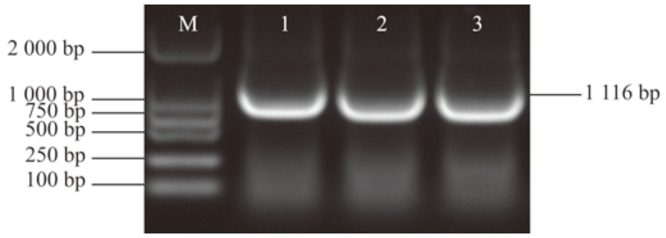

Figure S2. PCR cloning of *PfGPAT9* gene ORF. M, DNA Marker DL 2000; 1-3, The PCR product of full-length ORF of *PfGPAT9* gene.

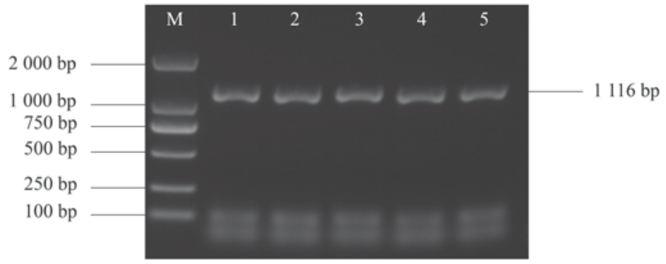

Figure S3. PCR detection of *PfGPAT9*- transgenic yeast. M, DNA Marker DL 2000; 1-5, INVSc1 transformed with pYES2.0+*PfGPAT9*.

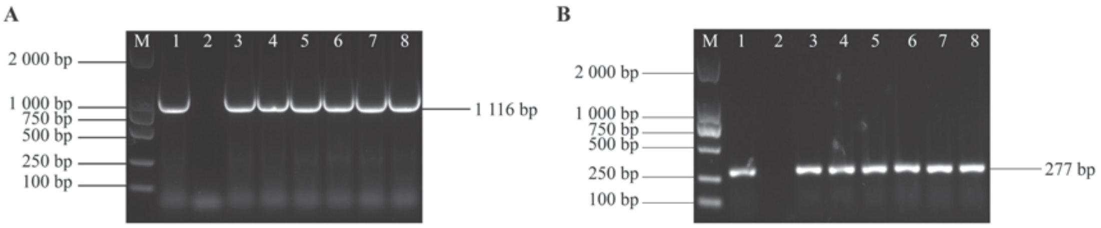

Figure S4. PCR detection of *PfGPAT9*- transgenic tobacco. PCR detection of genomic level **(A)** and transcription level **(B)** of *PfGPAT9*- transgenic tobacco lines. M, DNA Marker DL 2000; 1, pCAMBIA1303+*PfGPAT9* recombinant plasmid; 2, untransformed (WT) tobacco line; 3-8, *PfGPAT9*-transgenic tobacco lines.

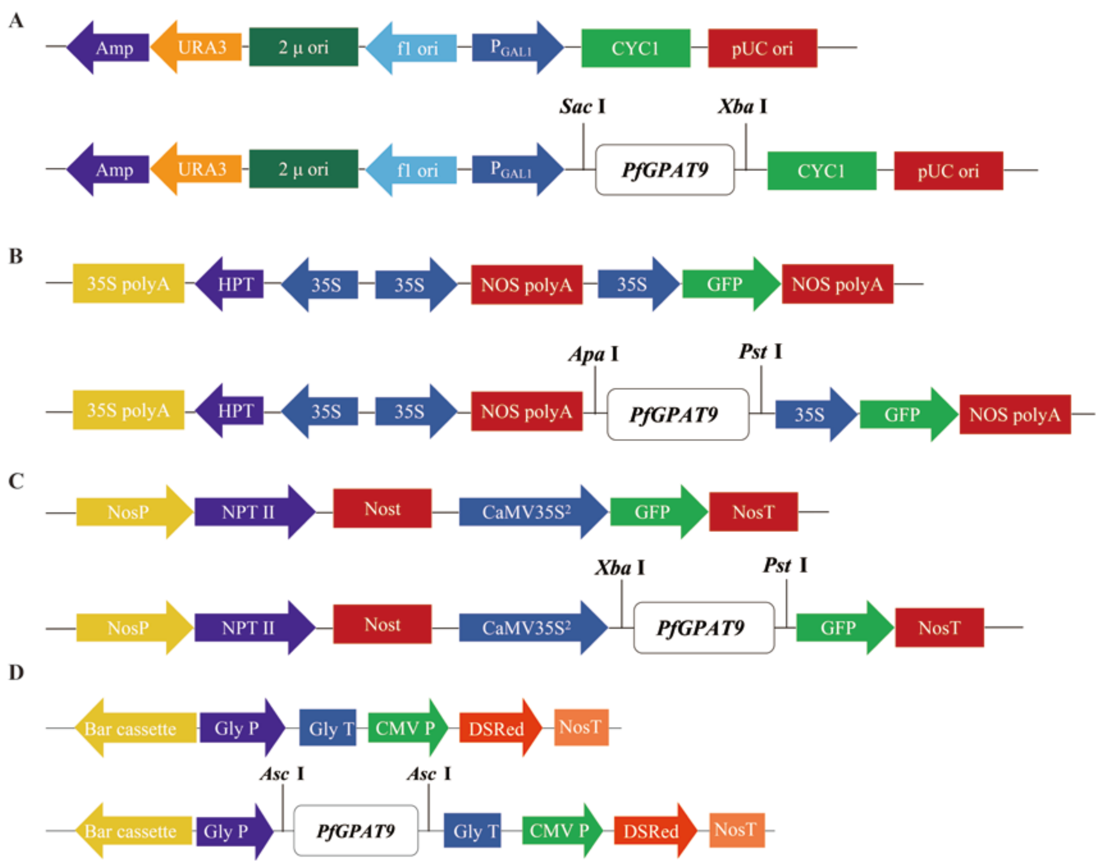

Figure S5. Diagrams showing different expression vectors carrying with the *PfGPAT9* gene. **(A)** Diagram showing pYES2.0+*PfGPAT9* yeast expression vector. **(B)** Diagram showing pCAMBIA1303+*PfGPAT9* plant expression vector. **(C)** Diagram showing pCAMBIA1300+*PfGPAT9*/GFP expression vector. **(D)** Diagram showing pJC-Gly-DSRB+*PfGPAT9* seed-specific expression vector.
